# Characterisation of the complete chloroplast genome of an endemic herb plant species, *Rheum palmatum* (Polygonaceae) in China

**DOI:** 10.1080/23802359.2019.1681319

**Published:** 2019-10-24

**Authors:** Xiu-Hang Zhang, Yu Long, Qing-Hua Yu, Tianyi Cao, Xin-Xin Chen, Jia-Ao Yu, Xin-Xin Gao

**Affiliations:** a Department of Burn Surgery, The First Hospital of Jilin University, Changchun, China;; b Department of Biological and Pharmaceutical Engineering, Wuhan, Hubei, China;; c Zhejiang Integrated Traditional Chinese and Western Medicine Hospital, Hangzhou, China

**Keywords:** *Rheum palmatum*, Polygonaceae, herb, chloroplast genome

## Abstract

*Rheum palmatum* has a long history in medicine, which is one of the main export medicinal herb in China. The complete chloroplast genome of *R. palmatum* was assembled and reported in this study. The *R. palmatum* chloroplast genome was 161,541 bp in length as the circular and consisted a large single-copy (LSC) region of 86,519 bp, a small single-copy (SSC) region of 13,112 bp and a pair of inverted-repeat (IR) regions of 30,955 bp. The nucleotide composition was asymmetric 31.2% A (Adenine), 31.5% T (Thymine), 19.0% C (Cytosine), and 18.3% G (Guanine) with an overall G + C content of 37.3%. It encoded 131 genes, including 86 protein-coding genes (76 PCG species), 37 transfer RNA genes (26 tRNAs species), and eight ribosomal RNA genes (four rRNAs species). The Phylogenetic relationships used neighbour-joining (NJ) method and the result showed that *R. palmatum* and *Rheum officinale* are phylogenetically related to each other in the family Polygonaceae. This study will be very important for Chinese medicinal herb research value and clinical drug development for future in China.

*Rheum palmatum* is the most important Chinese medicinal herb and belongs to the family Polygonaceae genus *Rheum*, which is one of the main export medicinal materials and has a long history in medicine in China (Li et al. 2007). It is grown as a vegetable for harvest of its edible leaf stalks, Chinese rhubarb is primarily grown as an ornamental for enjoyment of its huge rounded leaves and feathery plumes of summer flowers and a medicinal plant (Chang et al. 2014). *Rheum palmatum* has been used in Chinese medicine for treatment of a variety of medical conditions, including skin repair, constipation, diarrhoea, peptic ulcers, immunosuppression, high blood pressure, and cancer (Wen et al. 2018; Song et al. 2019). The research of *R. palmatum* was mainly concentrated on the active ingredients of medicines and less knowledge about the chloroplast genome and nuclear genome information were published.

*Rheum palmatum* was collected in The First Hospital of Jilin University in Changchun, Jilin, China (125.31E, 43.88 N). The total chloroplast DNA was extracted from of fresh stems and roots of *R. palmatum* using the modified CTAB method and stored at −80 °C in Department of Burn Surgery, The First Hospital of Jilin University (No.DBS-FHJU-01). Library construction was carried out using Illumina (Illumina, CA, USA) and was sequenced. FastQC Version 0.11.8 (Andrews 2015) was used to perform and remove low-quality reads and adapters for quality control. The chloroplast genome of *R. palmatum* was assembled and annotated using the MitoZ method (Meng et al. 2019). OrganellarGenomeDRAW Version 1.3.1 (Greiner et al. 2019) was used to draw the physical map of the chloroplast genome of *R. palmatum*.

The complete chloroplast genome of *R. palmatum* was 161,541 base pairs (bp) in length as the circular. The nucleotide composition was asymmetric 31.2% A (Adenine), 31.5% T (Thymine), 19.0% C (Cytosine), and 18.3% G (Guanine) with an overall G + C content of 37.3%. It comprised a characteristic quadripartite structure with a large single-copy (LSC) region of 86,519 bp, a small single-copy (SSC) region of 13,112 bp and a pair of inverted repeat (IR) regions of 30,955 bp. The chloroplast genome of *R. palmatum* was predicted to contain 131 genes, including 86 protein-coding genes (76 PCG species), 37 transfer RNA genes (26 tRNAs species), and eight ribosomal RNA genes (four rRNAs species). 18 species genes were occurred in double copies, including seven PCG genes species (*rpl2, rpl23, ycf2, ndhB, rps7, rps12*, and *ycf1*), seven tRNA genes species (*trnI-CAU, trnL-CAA, trnV-GAC, trnI-GAU, trnA-UGC, trnR-ACG*, and *trnN-GUU*), and four rRNA genes species (*rrn16, rrn23, rrn4.5*, and *rrn5*). All these 18 species genes were located in the IR regions. The complete chloroplast genome sequence of *R. palmatum* was submitted to the GenBank that NCBI accession Number is MK2627291.

To obtain *R. palmatum* phylogenetic position within the family Polygonaceae, the phylogenetic tree was constructed using the neighbour-joining (NJ) method. NJ tree analysis used the MEGA X (Kumar et al. 2018) and performed using 5000 bootstrap values replicate at each node. All of the nodes were inferred with strong support using the NJ methods. The final NJ phylogenetic tree was edited using the iTOL version 4.0 web (https://itol.embl.de/) (Letunic and Bork 2019). As shown in the NJ phylogenetic tree (Figure 1), *R. palmatum* and *R. officinale* (MH572012.1) are phylogenetically related to each other in the family Polygonaceae.

**Figure 1.**
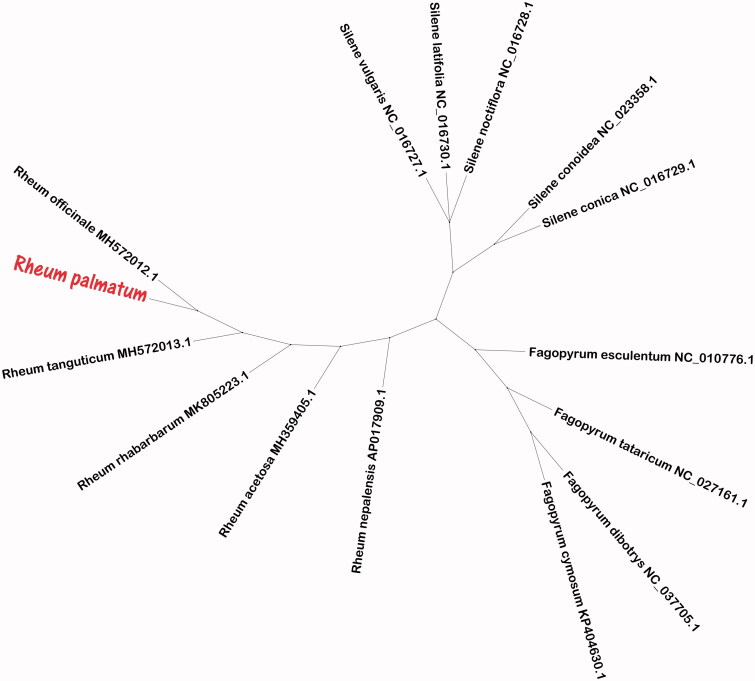
ML phylogenetic tree of *Rheum palmatum* with 15 species was constructed using chloroplast genome sequences. Phylogenetic relationships based on the neighbour-joining (NJ) method analysis using 5000 bootstrap replicates. Bootstrap support is indicated for each branch. GenBank accession numbers are given in figure.
